# Curvilinear Peri-Brainstem Hyperintense Signals as a Marker of Leptomeningeal Metastases in Lung Adenocarcinoma: A Multicenter Retrospective Case Series

**DOI:** 10.2174/0115734056383570250805110103

**Published:** 2025-08-08

**Authors:** Wangqiang Chen, Xian Ren, Guanmin Quan, Xuejun Zheng, Hongxin Jiang, Xiaokun Sun, Hui Zhang

**Affiliations:** 1 Department of Radiology, The Second Affiliated Hospital of Wenzhou Medical University, Wenzhou, Zhejiang, 325035, China; 2 Department of Neurology, The First Affiliated Hospital of Zhengzhou University, Zhengzhou. Henan, 450000, China; 3 Department of Radiology, The Second Affiliated Hospital of Hebei Medical University, Shijiazhuang, Hebei, 050000, China; 4 Department of Radiology, Linyi People's Hospital, Linyi, Shandong, 276000, China; 5 Department of Radiology, Gucheng County Hospital, Hengshui, Hebei, 053000, China; 6 Department of Radiology, The 92493 Hospital of the Chinese PLA, Huludao, Liaoning, 125000, China; 7 Department of Pharmacy, The Second Affiliated Hospital of Wenzhou Medical University, Wenzhou, Zhejiang, 325035, China

**Keywords:** Curvilinear hyperintense signal, Brainstem, Leptomeningeal metastases, Lung adenocarcinoma, CSF, FLAIR, MRI

## Abstract

**Introduction:**

Leptomeningeal metastasis (LM) is a severe complication of solid malignancies, including lung adenocarcinoma, characterized by poor prognosis and diagnostic challenges. This study assesses whether curvilinear peri-brainstem hyperintense signals on MRI are a characteristic feature of LM in lung adenocarcinoma patients.

**Methods:**

This retrospective study analyzed data from multiple centers, encompassing lung adenocarcinoma patients with peri-brainstem curvilinear hyperintense signals on MRI between January 2016 and March 2022. Clinical and radiological data were reviewed, and diagnostic and survival outcomes were analyzed.

**Results:**

Eleven patients (45-76 years; 6 males and 5 females) were included. The mean interval from lung adenocarcinoma diagnosis to detection of brainstem signal was 1.59 years. Four patients had elevated carcinoembryonic antigen levels, and 6 showed systemic metastases. MRI revealed curvilinear hyperintense signals along the pons, medulla, and cerebral peduncles on T2-Weighted Imaging (T2WI), Fluid-Attenuated Inversion Recovery (FLAIR), and Diffusion-Weighted Imaging (DWI). Mild linear enhancement was observed in 4 of 6 patients undergoing contrast-enhanced MRI, and tumor cells were detected in 4 of 6 Cerebrospinal Fluid (CSF) samples. The mean survival time in 7 patients with follow-up data was 3.42 months. Two patients exhibited peri-brainstem calcifications on CT 4–6 months after MRI and died shortly after.

**Discussion:**

These findings suggest that peri-brainstem curvilinear hyperintense signals and mild linear enhancement may serve as radiological markers of LM in lung adenocarcinoma. This pattern may reflect tumor infiltration or secondary changes in the leptomeninges.

**Conclusion:**

Peri-brainstem curvilinear hyperintense signals and mild linear enhancement on T2WI, FLAIR, and DWI may be characteristic of LM in lung adenocarcinoma. These findings suggest further evaluation with contrast-enhanced MRI or CSF analysis for confirmation.

## 1 INTRODUCTION

Leptomeningeal Metastasis (LM) is defined as the dissemination and proliferation of tumor cells along the brain and spinal meninges. It occurs in approximately 5–10% of patients with solid malignancies, most commonly lung cancer, breast cancer, and melanoma [1-3]. In Non-Small Cell Lung Cancer (NSCLC), the incidence of LM is approximately 3.4%, with a poor prognosis and a median survival time of 3-11 months [1, 4]. Early diagnosis of LM is crucial for enabling timely therapeutic interventions, which may improve survival outcomes.

However, the diagnosis of LM remains challenging, relying heavily on clinical findings, Cerebrospinal Fluid (CSF) cytology, and neuroimaging characteristics [1, 5, 6]. These factors are also considered predictive of treatment outcomes in LM patients [5]. Although CSF cytology is the gold standard for diagnosing LM, its sensitivity is low. In clinical practice, contrast-enhanced Magnetic Resonance Imaging (MRI) is often the preferred initial diagnostic tool for suspected LM cases. However, the diagnostic yield of MRI remains suboptimal, with false-negative rates as high as 30% [7].

Several case reports have described LM as presenting with abnormal findings on T2-Weighted Imaging (T2WI) and Fluid-Attenuated Inversion Recovery (FLAIR) sequences, particularly around the brainstem and cerebellar peduncles, even in the absence of contrast enhancement [8-10]. The abnormal MRI signals may result from leptomeningeal metastasis or microinfarctions [11]. To date, limited data are available regarding the association between these imaging findings and patient outcomes. This study aimed to improve the understanding and diagnostic accuracy of LM by analyzing the imaging features, clinical characteristics, and prognosis of 11 lung adenocarcinoma patients with curvilinear or band-like abnormalities around the brainstem.

## 2 MATERIALS AND METHODS

### 2.1 Clinical Data

This multicenter, retrospective observational study included eleven patients with lung adenocarcinoma from nine hospitals across China. Cases were selected from nine hospitals, including the Second Affiliated Hospital of Wenzhou Medical University, the Second Hospital of Hebei Medical University, the First Affiliated Hospital of Zhengzhou University, and Tianjin First Central Hospital, between January 2016 and March 2017. Inclusion criteria: (i) Pathologically confirmed lung adenocarcinoma, (ii) Presence of abnormal linear or curvilinear signals around the brainstem on Diffusion-Weighted Imaging (DWI) or FLAIR, with no or mild enhancement, and (iii) age ≥ 18 years. Exclusion criteria: (i) Absence of abnormal linear or curvilinear signals around the brainstem on DWI or FLAIR, (ii) poor image quality, or (iii) confirmed brainstem lesions caused by acute infarction, autoimmune encephalitis, or other non-tumor conditions.

Clinical data, including CSF cytology, symptom duration, time of tumor diagnosis, time to systemic tumor detection, presence of systemic metastases, tumor markers (*e.g*., Carcinoembryonic Antigen (CEA), and history of chemotherapy or radiotherapy, were recorded.

### 2.2 Imaging Analysis

MRI sequences included axial T1-Weighted Imaging (T1WI), T2WI, DWI (b=1000 s/mm^2^), sagittal T1WI, axial or coronal FLAIR (TI=2500 ms), and contrast-enhanced MRI. All images were independently reviewed by two neuroradiologists, each with over 10 years of clinical experience. In cases of disagreement, a consensus was reached through joint review. Lesions were assessed qualitatively. Signal intensity was classified as high or low relative to adjacent normal brain tissue. Linear or curvilinear signal abnormalities were defined as hyperintense signals along the brainstem surface or parenchyma, following anatomical contours, without mass effect. The presence of enhancement, concurrent lesions in other regions, and lesion evolution were also documented.

## 3 RESULTS

### 3.1 Clinical Characteristics

A total of 11 patients were included in this study, comprising 5 females and 6 males, aged 45 to 76 years, with a mean age of 59.09 ± 8.37 years. The most common clinical symptoms were dizziness (4/11), altered mental status or behavioral abnormalities (3/11), incoherent speech (2/11), headache (2/11), and gait disturbance (2/11) (Table **1**). Among the six patients who underwent CSF examination, tumor cells were detected in four cases. Elevated CEA levels were observed in four patients. Six patients presented with metastases, most commonly involving the liver and bone.

The average interval from the diagnosis of lung adenocarcinoma to the appearance of abnormal brain signals was 1.59 years, with the longest interval being six years. Four patients exhibited brainstem abnormalities at the time of their lung adenocarcinoma diagnosis. Only one patient underwent both chemotherapy and radiotherapy. Detailed clinical and laboratory data are presented in Table **1**.

### 3.2 Imaging Features

The imaging characteristics of all 11 patients are summarized in Table **2**. The lesions exhibited curvilinear hyperintensity on T2WI, FLAIR, and DWI along the pons, medulla, and cerebral peduncles. No significant abnormalities were detected on T1WI. Among the six cases with contrast-enhanced MRI, four showed mild linear enhancement adjacent to the brainstem. The pons was the most frequently affected region, being involved in all 11 cases, followed by the medulla in 8 cases and the cerebral peduncles in 7 cases. Hydrocephalus was observed in 2 patients.

### 3.3 Follow-up Outcomes

Seven patients were followed up, with an average survival time of approximately 3.42 months (range: 1–6 months). Among these, two patients developed linear calcifications around the brainstem at 4 and 6 months, respectively, as seen on Computed Tomography (CT), and both died shortly after the follow-up CT.

### 3.4 Representative Cases

A 59-year-old female (Case 1) was admitted with a 4-month history of dizziness and headache, accompanied by facial paralysis for 2 months. Brain MRI revealed curvilinear hyperintensities around the brainstem and cerebral peduncles on T2WI and FLAIR sequences Fig. (**1A**-**C**) and hypointensities on T1WI Fig. (**1D**). Contrast-enhanced MRI demonstrated linear enhancement around the brainstem and cerebellar sulci Fig. (**1E**, **F**). Based on the patient’s history of lung adenocarcinoma, the diagnosis of LM around the brainstem was established.

**Fig. (2):**
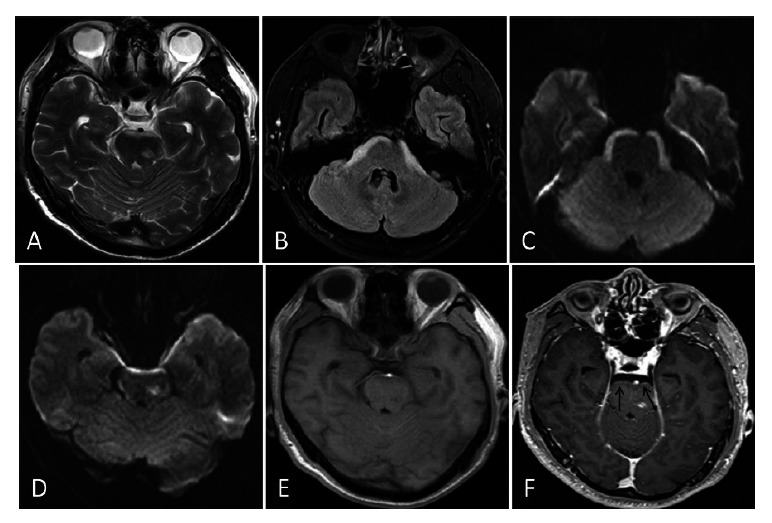
(Case 4): MRI shows arcuate hyperintensities along the brainstem on T2WI, FLAIR, and Diffusion-Weighted Imaging (DWI) sequences (**A**-**D**), with nodular hyperintensities in the cerebral peduncles. T1WI reveals corresponding arcuate and nodular hypointensities (**E**). Contrast-enhanced MRI (F, arrow) demonstrates mild linear and nodular enhancement around the brainstem.

A 54-year-old male (Case 4) presented with a 1-month history of headache. MRI showed arcuate hyperintensities along the brainstem on T2WI, FLAIR, and DWI sequences, with nodular hyperintensities in the cerebral peduncles Fig. (**2A**-**D**). Corresponding hypointensities were observed on T1WI Fig. (**2E**). Contrast-enhanced MRI revealed mild linear and nodular enhancement around the brainstem (Fig. **2F**, arrow). Based on the patient’s history of lung adenocarcinoma, the diagnosis of LM and brainstem metastases was confirmed.

**Fig. (3):**
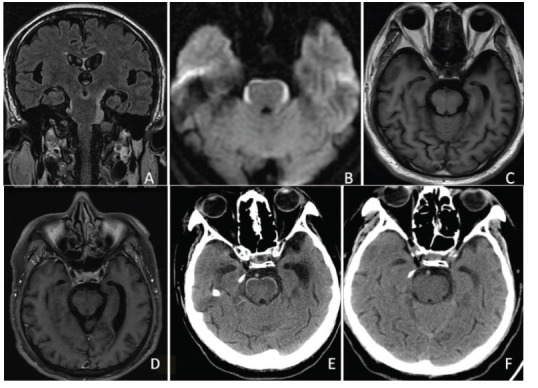
(Case 9): FLAIR and DWI sequences (**A**, **B**) reveal arcuate hyperintensities around the brainstem, with no significant abnormalities on T1WI (**C**). Contrast-enhanced MRI (**D**) shows no significant enhancement in the corresponding regions. Initial CT imaging did not detect arcuate calcifications around the brainstem; however, follow-up CT at 6 months (**E**, **F**) reveals arcuate high-density calcifications.

A 65-year-old male (Case 9) presented with a 15-day history of behavioral abnormalities. FLAIR and DWI sequences revealed arcuate hyperintensities surrounding the brainstem (Fig. **3A**, **B**), while no abnormalities were observed on T1WI (Fig. **3C**). Contrast-enhanced MRI showed no significant enhancement in the corresponding regions (Fig. **3D**). Initial CT imaging did not reveal calcifications; however, follow-up CT at 6 months demonstrated arcuate high-density calcifications around the brainstem (Fig. **3E**, **F**). The patient, diagnosed with LM around the brainstem, died shortly after the follow-up CT.

## 4 DISCUSSION

Our study highlights the diagnostic significance of curved high-intensity signals around the brainstem in T2WI, FLAIR, and DWI sequences, which are often underappreciated in routine practice. The findings in this cohort of 11 lung adenocarcinoma patients support the diagnosis of LM. First, tumor cells were identified in 4 out of 6 patients who underwent CSF analysis. CSF examination is the gold standard for diagnosing LM, and repeated lumbar punctures are necessary when LM is highly suspected [12]. Second, of the 6 patients who underwent contrast-enhanced MRI, 4 exhibited mild linear or curvilinear enhancement around the brainstem. The remaining 2 cases did not show enhancement but presented metastatic lesions in other brain regions. The variability in lesion enhancement may relate to different tumor growth patterns [12, 13]. In tumors with diffuse or non-fixed growth patterns, tumor cells freely float within the subarachnoid space without forming fixed tumor nests, resulting in absent or mild enhancement on MRI [10]. Conversely, nodular or linear enhancement typically corresponds to clustered tumor cells [12, 13]. Third, elevated serum CEA levels in 4 cases further support the diagnosis of active tumor progression.

The high signal intensity on T2WI, FLAIR, and DWI observed in this study aligns with findings in prior literature. This phenomenon may be attributed to microinfarcts caused by tumor invasion of perforating arteries, tumor-related syndromes, or chemotoxicity. Tumor cells likely infiltrate or deposit along the brainstem surface, which is richly supplied by perforating arteries from the basilar and superior cerebellar arteries [10]. The pons, as the most vascularized region of the brainstem, was the most commonly affected site in our cases. Additionally, tumor-induced microvascular occlusion may contribute to secondary infarction, further supporting the high signal intensity on DWI. The median time from MRI findings to LM diagnosis in this study was approximately 1.59 years, with 4 cases diagnosed simultaneously with lung adenocarcinoma. Only 1 patient received radiochemotherapy, underscoring the limited treatment options available. While tumor-related syndromes and chemotoxicity may contribute to imaging abnormalities, this study lacks robust evidence supporting such mechanisms. Additionally, potential imaging artifacts, such as those arising from CSF flow or vascular structures around the brainstem, were considered. However, CSF flow artifacts in the brainstem region are relatively rare and were not present in our cases. Moreover, the pulsation of small perforating arteries in this area is typically insufficient to cause motion-related artifacts comparable to those seen near larger vessels.

Calcification in brainstem metastases, observed in CT follow-ups of 2 cases, is reported here for the first time. The mechanisms of metastatic calcification may involve the phagocytosis of calcified scar tissue, dystrophic calcification of necrotic tumor areas, or tumor-secreted substances that promote calcification, particularly in adenocarcinoma [14, 15]. Calcified metastases were associated with poor prognosis, as both patients died shortly after CT revealed these changes. The significance of calcified brain metastases remains debated; some studies suggest they indicate longer survival when primary tumors are well-controlled, while others associate calcification with functional tumor activity and poor outcomes in lung adenocarcinoma [15]. We speculate that the unfavorable prognosis may be associated with impaired cerebrospinal fluid circulation due to extensive peribrainstem involvement, leading to intracranial hypertension. Nonetheless, the presence and prognostic implications of calcification in LM require further investigation through larger cohorts and pathological validation.

The predominant symptoms in this study included dizziness (4/11) and altered mental status (3/11), with other symptoms such as headache, confusion, and cranial nerve deficits also reported. Symptom manifestation correlated with tumor location, with brainstem and cerebral peduncle involvement leading to corresponding neurocognitive and functional impairments. Evaluating such symptoms is crucial for assessing the risk of LM in systemic malignancies. Up to 20% of patients with neurological symptoms are diagnosed with LM post-mortem, while 5–8% may remain asymptomatic [6, 16]. In suspected cases of brain metastasis, an enhanced MRI or CSF examination should be performed regardless of symptomatology to confirm the diagnosis.

Although this study suggests a potential diagnostic value of curvilinear peribrainstem hyperintensity on T2WI, FLAIR, and DWI in patients with LM, several limitations must be acknowledged. The retrospective design and small sample size (n = 11) inherently introduce biases, including variability in clinical management, imaging protocols, and incomplete clinical or imaging data. Additionally, the CSF cytology positivity rate in this cohort may reflect sampling variability and should be interpreted cautiously. Rather than proposing definitive diagnostic criteria, this study aims to draw attention to an uncommon imaging manifestation of LM. Given the known low sensitivity of CSF cytology, we suggest incorporating this imaging pattern into a multimodal diagnostic framework, including repeated CSF analysis, clinical assessment, and, where available, emerging liquid biopsy tools, such as CSF ctDNA and circulating tumor cells [17, 18]. According to the 2024 Neuro-Oncology (SNO) and American Society of Clinical Oncology (ASCO) consensus [17], these biomarkers may enhance diagnostic yield and guide treatment decisions.

Clinically, the detection of this pattern should prompt early multidisciplinary involvement and consideration of intensified treatment strategies, particularly in patients with additional poor prognostic indicators, such as lesion calcification. The average survival time of 3.42 months observed in this cohort is provided as a descriptive reference rather than a prognostic benchmark, given the influence of unmeasured factors such as age, tumor type, treatment, and performance status. Notably, while this imaging manifestation appears uncommon in non-neoplastic or other neurological conditions based on our clinical experience, its specificity remains to be determined. Future prospective studies with larger cohorts, standardized imaging protocols, and broader differential diagnostic comparisons are warranted to validate these findings, elucidate their prognostic relevance, and determine whether early intervention based on this imaging marker can improve clinical outcomes.

## CONCLUSION

In conclusion, the presence of abnormal high-intensity signals around the brainstem on T2WI, FLAIR, and DWI, coupled with linear or curvilinear enhancement on contrast imaging, aids in diagnosing LM. Calcification of LM lesions indicates a poor prognosis. Contrast-enhanced MRI or CSF analysis should be performed when brain metastasis is suspected, regardless of the presence of clinical symptoms.

## Figures and Tables

**Fig. (1):**
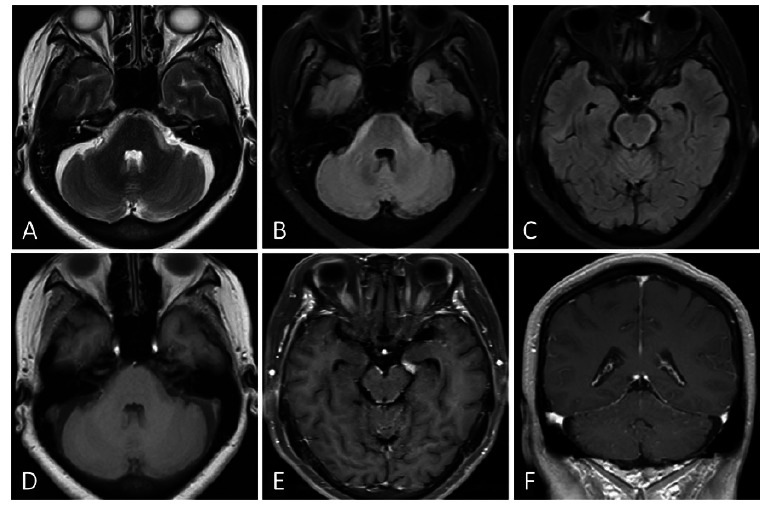
(Case 1): MRI demonstrates curvilinear hyperintensities around the brainstem and cerebral peduncles on T2-Weighted Imaging (T2WI) and Fluid-Attenuated Inversion Recovery (FLAIR) sequences (**A**-**C**) and hypointensities on T1-Weighted Imaging (T1WI) (**D**). Contrast-enhanced MRI (**E**, **F**) reveals linear enhancement along the brainstem and cerebellar sulci.

**Table 1:** Clinical and laboratory data of 11 patients.

**Case No.**	**Gender**	**Age**	**Medical history (Duration, years)**	**Metastasis to other sites**	**Chemotherapy/ Radiotherapy**	**CSF (Tumor cells)**	**CEA (ng/ml)**	**Clinical Symptoms (Duration)**				**Survival Time Post-MRI**
								**Dizziness**	**Headache**	**Nonsense**	**Other**	
1	F	59	LA (0#)	-	-	+	32	4 M	4 M	0	Facial Paralysis (2 M)	Death (1 M)
2	M	76	LA (0#)	-	-	+	381	4 M	0	0	Mental disorder (2 M)	Death (1 M)
3	F	61	LA (0#)	-	-	+	Unknown	0	0	3 h	0	Death (6 M)
4	M	54	LA (3)	+	+	Unknown	Unknown	0	1 M	0	0	Death (4 M)
5	M	60	LA (0#)	+	-	Unknown	Unknown	1 M	0	0	Hearing loss (1 M)	0
6	F	53	LA (1)	+	-	Unknown	1500	0	1 W	0	0	0
7	F	58	LA (4)	-	-	+	Unknown	0	0	0	Behavioral abnormalities (10 d)	0
8	M	67	LA (1)	-	-	-	113.49	20 d	0	0	Unsteady gait (20 d)	0
9	M	65	LA (6)	+	-	-	Unknown	0	0	0	Behavioral abnormalities (15 d)	Death (6 M)
10	F	52	LA (2)	+	-	Unknown	Unknown	0	10 d	0	0	Death (4 M)
11	M	45	LA (0.5)	+	-	Unknown	Unknown	0	0	2 M	Unsteady gait (2 M)	Death (2 M)

**Note:** M: Male, F: Female, LA: Lung Adenocarcinoma, M: month, W: week, d: day. “0”: No such symptoms or no follow-up. “0#”: Simultaneous diagnosis of lung adenocarcinoma and discovery of high signal around the brainstem.

**Table 2:** Imaging characteristics of 11 patients.

-	1	2	3	4	5	6	7	8	9	10	11
**T1WI (low signal)**	+	+	+	+	+	+	+	+	+	+	+
**T2WI (high signal)**	+	+	+	+	+	+	+	+	+	+	+
**FLAIR (high signal)**	+	+	+	+	+	+	+	+	+	+	+
**DWI (high signal)**	+	+	+	+	+	+	+	+	+	+	+
**Lesion enhancement**	+	+	-	+	N	N	N	N	-	N	+
**Cerebral peduncle**	+	+	-	+	-	-	+	+	+	-	+
**Pons**	+	+	+	+	+	+	+	+	+	+	+
**Medula oblongata**	+	+	-	+	-	+	+	+	+	-	+
**Cerebellum**	+	-	-	-	-	-	-	-	-	-	+
**Cerebrum**	-	+	-	-	-	-	-	-	-	-	-
**Hydrocephalus**	-	+	-	-	-	+	-	-	-	-	-

N: No contrast enhancement data.

## Data Availability

All data generated or analyzed during this study can be obtained from the corresponding author [H.Z] upon reasonable request.

## LIST OF ABBREVIATIONS

LM: Leptomeningeal Metastasis
NSCLC: Non-Small Cell Lung Cancer
CSF: Cerebrospinal Fluid
T2WI: T2-Weighted Imaging
FLAIR: Fluid-Attenuated Inversion Recovery
CEA: Carcinoembryonic Antigen
ASCO: American Society of Clinical Oncology

## STANDARDS OF REPORTING

STROBE guidelines were followed.
